# A study on the relationship between flood safety awareness and vulnerability/resilience

**DOI:** 10.1016/j.heliyon.2024.e39819

**Published:** 2024-10-26

**Authors:** Kiyong Park, Seol A. Kwon

**Affiliations:** a Dept. of Human Resource Development, LX Education Institute, Korea Land and Geospatial Informatix Corporation, Chungnam, 32522, Republic of Korea; b National Crisisonomy Institute, Chungbuk National University, Cheongju, 28644, Republic of Korea

**Keywords:** Disaster resource management, Natural disaster, Structural vulnerability, Social resilience, Flood safety awareness

## Abstract

This study aimed to investigate the relationship between flood safety awareness and the vulnerability/resilience associated with flood damage, focusing on the characteristics of regions that have experienced floods. Specifically, this study addresses the following research questions: (1) How does flood safety awareness vary among regions with different levels of vulnerability and resilience? (2) What patterns emerge in the relationship between regional characteristics and residents' flood safety awareness? A fuzzy analysis was used by selecting structural and social indicators to analyze vulnerability and resilience. A survey was conducted for each region to assess flood safety awareness among residents. The analysis results showed that vulnerability and resilience exhibit three distinct patterns according to regional characteristics, which correspond to the flood safety awareness of residents. Regions with low vulnerability and high resilience demonstrated high safety awareness, whereas regions with high vulnerability and low resilience demonstrated low safety awareness. This study lies in its comparative analysis of flood safety awareness across different regional contexts and its use of fuzzy analysis to provide a nuanced understanding of vulnerability and resilience. This study accurately identifies problems and regional differences by analyzing vulnerability and resilience and establishing preventive measures that fit regional characteristics. Furthermore, this study provides a guideline for suggesting effective disaster response strategies, contributing new insights into disaster preparedness and community resilience.

## Highlights

• Effective disaster response strategies are able to immediately proposed for regions that have experienced flood.
• Exploring the relationship between structural vulnerability and social resilience within flood damage based on the characteristics of regions.
• Analysis of vulnerability and resilience can be applied to check the status of region residents' flood safety awareness.
• High-vulnerability and low-resilience regions are supposed to be selected as well as systematic response is possible through reasonable decision-making.

## 1 Introduction

The climate crisis has increased the frequency and intensity of natural disasters worldwide, posing significant challenges to urban resilience and disaster management [1,2]. Natural hazards, exacerbated by climate change, negatively impact various aspects of life, and despite global efforts to address these environmental issues, the outcomes remain uncertain [[3], [4], [5]]. Resilience, a crucial term in disaster management, refers to the ability to "bounce back" and return to a pre-disturbance state after experiencing disruptions caused by risk factors [[6], [7], [8]]. This concept encompasses the extent of interactions between humans and the environment [9,10]. According to the UNISDR, resilience is the ability of a system, community, or society exposed to risks to resist, absorb, and recover from their impacts [11,12]. In disaster management, resilience specifically entails network capacity [13,14], livelihood capital [[15], [16], [17]], physical systems [[18], [19], [20]], and governance [[21], [22], [23]].

Natural disasters are often examined through the lens of structural vulnerability, which is particularly critical as over 50 % of the global population resides in cities [24]. Structural characteristics significantly influence the magnitude of damage caused by natural disasters in urban areas [25]. Mileti (1999) demonstrated that the potential for floods, wind damage, and tsunami damage varies depending on building characteristics and structure. The concept of resilience has been explored from various perspectives, including engineering resilience, which focuses on the speed of recovery, ecological resilience, which considers environmental interactions, and evolutionary resilience, which emphasizes adaptive management. Additionally, critical resilience addresses social inequalities in flood risk management [[130], [131], [132]].

Despite extensive studies on social resilience and structural vulnerability, there are few that have examined the correlation between these two factors [[26], [27], [28]]. Emrich et al. (2020) highlighted that socially disadvantaged groups and minority households received less financial and recovery support despite severe damages. Other studies have demonstrated that financial support is often inaccessible to minority and low-income groups, indicating that financial and recovery support systems fail to consider the response capabilities of socially vulnerable populations. However, previous research has primarily focused on financial support as a variable without sufficiently exploring the broader correlation between social resilience and structural vulnerability [[26], [27], [28]].

This study aims to fill this gap by identifying regional disaster response capabilities through investigating the relationship between structural vulnerability and social resilience in the context of natural disasters, particularly flood damages. Additionally, it seeks to develop effective response measures by comparing regional variations in practical disaster response capabilities and flood safety awareness using the flood safety index perceived by residents. By analyzing financial and spatial data along with residents' risk awareness, this study aims to understand the complex interactions between structural vulnerability and social resilience and propose strategic and comprehensive disaster response measures.

The objective is to contribute to the rich body of literature on flood risk management by integrating insights from various perspectives on resilience. The study will draw on seminal works such as those by Davoudi (2012), who discusses the different resilience concepts critical for flood risk management and local communities [130], and Laeni et al. (2019, 2020, 2021), who provide valuable insights into the institutional and policy frameworks that enhance urban flood resilience [[29], [30], [31]]. Additionally, the study will reference relevant literature in flood risk management and resilience, including works by Busscher et al. (2019), Edelenbos et al. (2017), and Forrest et al. (2019, 2020), to ensure a comprehensive understanding of the theoretical and practical aspects of resilience in urban settings [39,[133], [134], [135], [136]].

By addressing these gaps and building on existing research, this study aims to provide a robust framework for understanding and enhancing the resilience of urban areas to flood risks, ultimately contributing to more effective and equitable disaster management strategies.

## 2 Methods

### 2.1 Theoretical discussion on structural vulnerability and social resilience

An analysis on structural vulnerability either examines the characteristics of an individual building or appropriately classifies the collective characteristics of a building. Analyzing collective building characteristics is often appropriate for overall disaster management of urban regions [32]. Previous studies have specified deterioration, underground area, floor area ratio, and material as general variables for analyzing structural vulnerability [[33], [34], [35]]. Europe applies the Risk-UE project and the United States of America follows HAZUS (FEMA-NIBS 2003), which consider construction material, building height, and design methods for analyzing structural vulnerability [[36], [37], [38]].

These methods incorporate probabilistic models and specific disaster scenarios, providing comprehensive risk assessments for urban disaster management [138]. Recent advancements also integrate machine learning and AI to predict and analyze structural vulnerabilities, emphasizing the role of advanced technologies in improving disaster response and resilience [139−140].

Social resilience is related to “structural violence,” defined as “direct and indirect violence built by suppressed social order” affecting the self-realization of humans and pursuing safe life [141]. Weak social resilience leads to political and economic neglect, causing individuals to powerlessly accept their circumstances within the violence of unstable life [[39], [40], [41]]. Drakes et al. (2021) discussed social resilience, which focuses on the non-physical needs of humans, such as social, psychological, and community aspects. It helps measure human sensitivity to disaster impact. Measuring social resilience through the socially vulnerable class helps explain the influence and relationship of disasters including fatality [42], economic loss [43], response capability [44,45], and public resources [46] in disasters. Furthermore, social resilience encompasses community networks, social capital, and governance, which influence disaster preparedness and recovery [142]. Case studies from the Philippines show how frameworks assess vulnerability using community-based data, expert inputs, and participatory activities, providing valuable insights for disaster risk reduction [143].

### 2.2 Deducing evaluation items

Certain indicators including underground building area, extent of deterioration, construction material, and floor area ratio have been selected to objectively evaluate structural vulnerability and social resilience concerning urban flooding (see Table 1). Buildings with a large underground area or many underground levels have a high possibility of flooding as water can easily penetrate underground structures. Water inflow through closed door or windows cannot be completely blocked. Consequently, flood damage possibility increases as water can quickly flow into underground structures [47]. An underground area also affects the drainage system as underground structures without an effective drainage system can be easily submerged. However, any drainage system can be overloaded or may not operate properly during flooding caused by heavy rain. Water cannot be easily discharged from underground structures in such case [48]. Thus, flood damage can increase for buildings with underground levels or large underground area. Electrical facilities, mechanical devices, and storage items in underground structures can also be flooded, leading to secondary damages. Cost and time required for recovery and repair may increase as well [49]. Another issue concerns evacuation difficulty in case of floods. Emergency evacuation from the underground levels of a building tend to be difficult as underground floors submerge when flood elevation increases [50]. Therefore, buildings with a large underground area or many underground levels generally have a high risk of floods, thus being vulnerable to disasters. These factors must be considered when designing buildings or managing flood risks.

**Table 1:** Basis of evaluation items.

	Indicator	References	Data source
Vulnerability of structural	Underground space	Schinke et al. (2012); Colven (2020); Rosenzweig et al. (2019); Rosenzweig et al. (2018)	National Spatial Data Infrastructure Portal
	Building deterioration	Ouma and Tateishi (2014); Rimba et al. (2017); Chang and Huang (2015)	
	Building material	Stephenson and D'ayala (2014); Qasim et al. (2017); Membele et al. (2020); D'Ayala et al. (2020)	
	Floor area ratio	Fatemi et al. (2020); Miranda and Ferreira (2019); Ishaya et al. (2009)	
Resilience of Social	Land value	Pagano et al., 2020; Cutter (2016); Islam and Walkerden (2022); Rezvani et al. (2023); Siebeneck et al. (2015); Kapucu et al. (2013)	
	Working age population	Tagliacozzo et al. (2021); Tuitjer (2019); Sanfelici (2021); Guadagno (2020); Shaw et al. (2010); Prashar and Shaw (2012); National Research Council and Geographical Sciences Committee (2011); Twigg (2009)	National Geographic Information Institute
	Distance to medical and rescue facilities	Zhong et al. (2014); Albanese et al. (2008); Zhong (2014); Egawa et al. (2018)	
	Permeable area	Lule-Hurtado (2015); MS et al. (2022); Gavin (2017); Zhang et al. (2019); Liu et al. (2021); Galderisi and Treccozzi (2017); Tyler (2016)	Environmental Geographic Information Service

Buildings with a high floor area ratio commonly increases the ground surface impermeability rate. In other words, rainwater does not permeate through the ground if buildings, roads, and artificial surfaces increase. The risk of floods increases as water quickly covers the ground surface under heavy rain [51]. A large-scale drainage system must be constructed to prevent such damages, but implementing such system is practically impossible. Without an effective large-scale drainage system, an urban region can be flooded during heavy rain [52]. From the perspective of flood response, damage recovery and repair become complicated and time-consuming, while more houses get damaged along with impaired urban functions [53]. Therefore, sensitivity to flood damage increases as the building floor area ratio increases due to ground surface impermeability, difficulty in utilizing urban infrastructure and functions, limited drainage systems, and challenges in evacuation and response.

Building material is an element that determines the vulnerability to flood [54]. Steel frame and concrete are commonly known as sturdy materials that can effectively block water during floods and minimize structural damages, thus exhibiting high stability [55]. In contrast, wooden and round timber structures are more vulnerable to floods. Timber may rot or expand when exposed to water, which ultimately deforms or breaks due to degraded structural stability [56,57].

Deteriorated buildings may display structural defects over time. When buildings with such defects experience floods, structural strength is lowered and cracks can be generated. Thus, deteriorated buildings are highly likely to experience structural damages during floods [58]. Water can be quickly permeated or absorbed in deteriorated buildings due to defective permeability of exterior materials. Thus, flood damages may propagate fast or structures may become unstable. Moreover, deteriorated buildings may lack up-to-date drainage systems. It implies that flooding can happen quickly inside the structure because water is permeated or cannot flow smoothly during floods [59,60].

Indicators for analyzing social resilience include appraised land value, working age population, accessibility to hospitals and fire stations, and permeable area, which have been selected based on the following grounds.

Appraised land value indicates the value of land and determines most of individual or household assets [61,62]. In general, more assets raises financial stability, which eventually enables a person or a household to receive financial support or urgent money in case of disasters. Accordingly, individuals or households with more assets receive and utilize basic and necessary supports or services efficiently in a relatively shorter time period during disasters [63,64]. Individuals or households with more assets are also more likely to have formed a wide social network. A strong connection with communities facilitates receiving necessary supports faster [65], and allows selecting various response strategies such as purchasing essential services or hiring workforce in disaster situations [66]. Specifically, appraised land value represents asset size, thus playing a crucial role in improving resilience by planning and executing various response strategies after a disaster occurs [61,62]. Therefore, high appraised land value leading to high disaster resilience emphasizes the positive correlation between asset size and resilience, and can help individuals or households recover in disaster situations.

Working age population tends to have higher resilience than the social disadvantaged class, which can be considered non-working age population [67]. Particularly, temporary workers, children, elderly, and specially abled people, typically classified as the non-working age population, are highly vulnerable during disasters. For example, temporary workers do not often experience stable working environment and have a limited social safety net. Thus, they have economic rigidity and lack experience to efficiently utilize available resources, thus having low resilience after disasters [68]. Children, elderly, and specially abled people highly depend on others for survival and have low resilience due to relatively poorer physical abilities [69,70]. Contrarily, working age population have regular jobs, enabling them to accumulate financial resources [71]. Working age population is more socially active and may be in an advantageous position to building networks. More networks form an environment where individuals can promptly obtain required support or information in case of disasters [[72], [73], [74]]. Working age population also comes from different educational and occupational backgrounds and is equipped with relevant knowledge and skills. It indicates that they can apply creative and appropriate methods to solve problems and adapt to disaster situations [73]. In general, working age population holds regular jobs or sufficient assets, which create a stable life and ultimately help them satisfy daily needs during a disaster.

If medical and rescue facilities such as hospitals or fire stations are closely located and easily accessible, injured persons can promptly receive first aid during disasters. Fire and rescue personnel can also be quickly dispatched to engage in lifesaving efforts [75]. Close vicinity to hospitals and fire stations allows relevant information to be quickly spread and corrected. Important information can be promptly obtained during disasters, while the function and role of medical and rescue resources can be utilized effectively [76]. It allows residents to feel safe and trust the supportive sources, and lowers social anxiety to help residents cooperate in disaster situations [77,78].

On lands with a large permeable area, ground surface water is easily absorbed into the ground during heavy rain or flood. Consequently, the discharge capacity improves [[79], [80], [81]], thus lowering flood damages through effective natural disaster response capability [82,83]. It also allows the ecosystem to be resilient and recover from natural disasters [84,85]. Therefore, lands with a large permeable area can quickly recover and respond during disasters by adequately managing water flow and reducing flood damages.

### 2.3 Target area

**Fig. 1:**
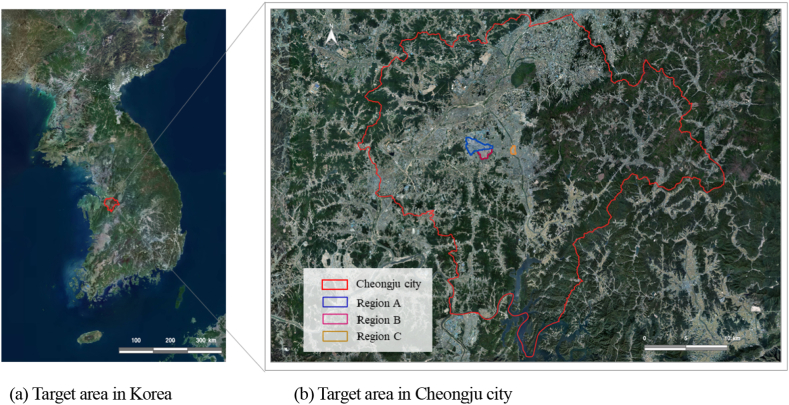
Target area.

The target area is as shown in Fig. 1. The City of Cheongju in Korea has an administrative area of 7406.96 km^2^ and population of 1,597,427 (see Fig. 1a) [86]. Three major flood-prone areas in Cheongju were selected as the study target area (see Fig. 1b).

On July 16, 2017, 90 mm rain per hour was recorded in Cheongju, while on August 10, 2022, a rainfall of 235 mm caused serious damages. Particularly, underground parking lots and roadsides in regions A and B were flooded in addition to the damage to a transformer vault, which disrupted water and electricity supply. The residents and merchants scooped out rainwater using buckets to prevent underground parking lots from being flooded, and stacked sandbags near the entrances to minimize flood damages [87]. Region C encompasses residential and commercial areas formed in lowlands along the riverside. It is most vitalized urban region in Cheongju, but inundation and flood damages occur repeatedly due to urbanization [88].

### 2.4 Analysis method

Structural vulnerability and social vulnerability were examined to analyze regional disaster response capabilities. The flood risk awareness of residents was analyzed using a survey.

The most widely used methodology for measuring vulnerability and resilience is creation of an index from several variables and indicators [89,90]. Since different indicators have unique characteristics, their impact on vulnerability and resilience differs. Thus, the relative importance of each indicator or item can be expressed numerically [91]. The fuzzy theory is employed to numerically and logically express ambiguity in the language and thinking of humans. It is extremely challenging to numerically express vulnerability and resilience associated with flood damages, where the characteristics of each indicator vary. Therefore, a fuzzy inference system was applied to numerically express the relation between vulnerability and resilience as well as their impacts, which is a linguistic function of humans.

Fuzzy set theory has been developed and extensively applied since 1965 [92]. Fuzzy logic is a multi-value logic that quantifies uncertain statements [93]. Since it was designed to supplement the uncertainty analysis of wide-ranging information without clear boundaries, these key causes are properly explained through fuzzy membership functions [94]. Fuzzy classification consists of an n-dimensional tuple of membership degrees, which describes the degree of class assignment μ of the considered object obj to the n considered classes [7,138].

(1) fclass,j=[μclass1(obj),μclass_2(obj),…μclass_n(obj)]

Crisp classification would only provide information on which membership degree is the highest, whereas this tuple contains all information about the overall reliability, stability, and class mixture. Fuzzy inference generally consists of three main steps: fuzzification, fuzzy inference rule and defuzzification. During the first stage, fuzzification, the value of the input variable measured with a single clear value is replaced with the appropriate fuzzy value. In the second stage, fuzzy inference rule, a rule is derived using the number of possible cases as conditional statements and the actual conditional statements. The third step, defuzzification, converts the fuzzy value defined in the entire set of outputs into an identifiable fuzzy value [95].

For analyzing the extent at which the residents feel safe from flood risks according to regional characteristics based on the social safety awareness survey developed by Korea Statistics (KSTAT), 100 surveys were given out in each region (300 surveys in total) for 30 days from June 1 to 30, 2023. For deducing a safety index, the Likert scale questions, which enable the residents to self-evaluate their own flood safety awareness level, were devised into categories of daily risks, financial conditions, living safety, health conditions, and residential environment. The Likert scale questions were used owing to the methodological validity [96], objectivity in measuring opinions and attitudes, and ability to convert human behavior into statistics [97].

## 3 Results and discussion

### 3.1 Data construction and fuzzification

A total of 4994 data pieces collected from the National Spatial Data Infrastructure Portal, National Geographic Information Institute, and Environmental Geographic Information Service with respect to each indicator are organized by lot number Table 2, Table 3. To facilitate the analysis process, the cell was divided into 100 m × 100 m units, and then merge and split operations were performed to create 515 cells.

**Table 2:** Data for structural vulnerability.

# of items	Underground space (㎡)	Building deterioration	Building material	Floor area ratio (%)
1	173.98	24	2	225.12
2	0.00	22	2	294.73
3	0.00	23	2	239.95
4	173.97	22	2	298.71
5	0.00	22	2	223.25
6	141.56	23	2	173.89
7	143.60	22	2	177.21
:				
:				
4991	254.34	23	1	368.45
4992	0.00	16	2	176.78
4993	0.00	11	2	179.06
4994	0.00	15	2	203.08

**Table 3:** Data for social resilience.

# of items	Land value (won/㎡)	Working age population	Distance to medical and rescue facilities (㎞)	Permeable area
1	906500	106	1.833	1
2	1745000	184	1.833	1.5
3	988000	135	1.833	1
4	1760000	164	2.002	1
5	1920000	103	2.002	1
6	2300000	34	2.097	1
7	2300000	25	2.097	1
:				
:				
4991	799000	37	1.685	1
4992	668000	41	1.685	1
4993	386500	61	1.685	1
4994	601000	31	1.685	1

Fuzzification was applied based on the standard-distribution method; since the measurements of each indicator differed, the values were classified according to set criteria and normalized between 0 and 1, which indicate extremely low and extremely high vulnerability and resilience, respectively. The normalized values represent vulnerability and resilience level of each region based on structural or social standards. The normalization method is as shown in Equation (2) [98].

(2) $$b_{i}=\frac{a_{i}-a_{\min}}{a_{\max}-a_{\min}}$$

### 3.2 Fuzzy inference rules: generating 81 rules

For explaining the fuzzy inference rules, this study conducted an expert interview for deducing each indicator weights and membership function. The membership function can be derived by standardizing each indicator value Table 4, Table 5. A total of 81 fuzzy inference rules were set for each situation of vulnerability and resilience.

**Table 4:** Determine the membership function of structural vulnerability.

	Underground space			Building deterioration			Building material			Floor area ratio		
	Minimum	Average	Maximum	Minimum	Average	Maximum	Minimum	Average	Maximum	Minimum	Average	Maximum
Low	0.00	0.01	0.05	0.00	0.14	0.28	0.00	0.25	0.45	0.00	0.01	0.22
Moderate	0.03	0.08	0.24	0.23	0.42	0.47	0.38	0.63	0.75	0.12	0.16	0.37
High	0.19	0.58	1.00	0.38	0.78	1.00	0.70	0.88	1.00	0.30	0.65	1.00

**Table 5:** Determine the membership function of social resilience.

	Land value			Working age population			Distance to medical and rescue facilities			Permeable area		
	Minimum	Average	Maximum	Minimum	Average	Maximum	Minimum	Average	Maximum	Minimum	Average	Maximum
Low	0.00	0.11	0.22	0.00	0.01	0.04	0.00	0.23	0.56	0.00	0.25	0.55
Moderate	0.16	0.25	0.41	0.02	0.13	0.47	0.37	0.63	0.83	0.50	0.63	0.75
High	0.33	0.64	1.00	0.35	0.62	1.00	0.69	0.89	1.00	0.65	0.88	1.00

Example: If (underground space is high) and (high deterioration) and (building material is high) and (floor area ratio is high), then (weight is highly vulnerable).

**Fig. 2:**
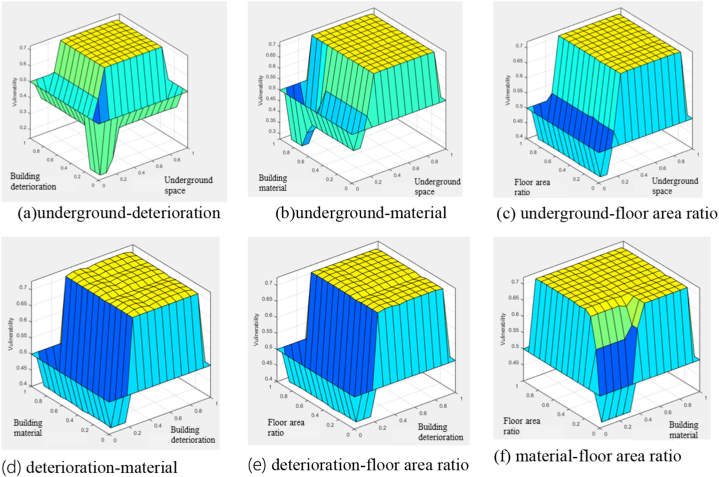
Scheme of structural vulnerability.

**Fig. 3:**
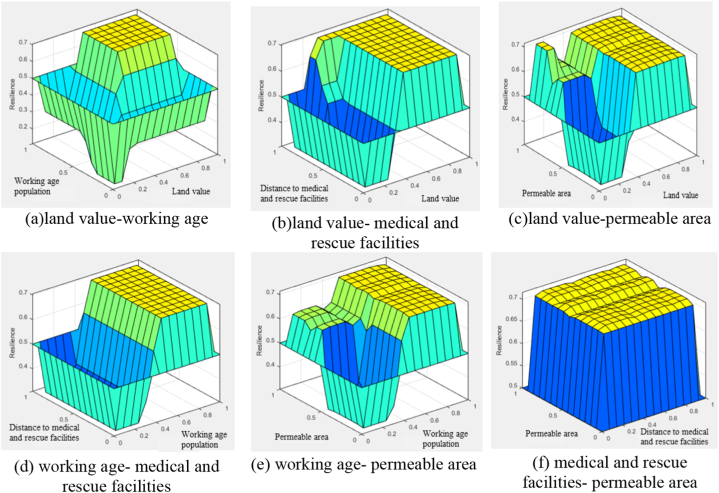
Scheme of social resilience.

For measuring relative importance of evaluation items, the membership function was graphed and the fuzzy inference was applied Fig. 2, Fig. 3. As a result of the vulnerability analysis, the importance of underground spaces is the highest, followed by deterioration, building material, and floor area ratio (Fig. 2a–f). Underground spaces are directly affected by flooding, and without an adequate drainage system, the damage can be significantly exacerbated. Additionally, secondary damage must be considered. Deterioration is highly important because it signifies structural defects in buildings. Building material and floor area ratio are also important factors, but in modern urban settings, wooden structures are rare. The inconvenience related to urban functional use represents indirect damage, which is why these factors are relatively less important.

As a result of the resilience analysis, the importance of appraised land value is the highest, followed by working-age population, distance to medical and rescue facilities, and land cover (Fig. 3a–f). Appraised land value is a key indicator of the asset status in the area, facilitating easy funding. A higher-working age population implies sufficient assets, meaning the area can quickly return to its original state after a disaster. Being close to an emergency facility means we can receive quick emergency treatment. Permeable areas have significant long-term preventive and recovery effects. However, considering that flood damage generally results in fewer casualties and factoring in time, its relative importance is deemed lower.

### 3.3 Defuzzification

The approximate inference results based on fuzzy inference were output as a fuzzy set and subjected to the defuzzification process, which expresses the set as a clear numerical value. This centroid method was used for this procedure and the defuzzification process is described in Equation (3).

(3) $$\begin{array}{cc} Z_{0}= & \frac{\int \mu_{c}\left(z\right)\cdot zdz}{\int \mu_{c}\left(z\right)dz}or \end{array}Z_{0}=\frac{\sum_{i=0}^{n}\mu_{i}\mu \left(u_{i}\right)}{\sum_{i=0}^{n}\mu \left(u_{i}\right)}$$

**Fig. 4:**
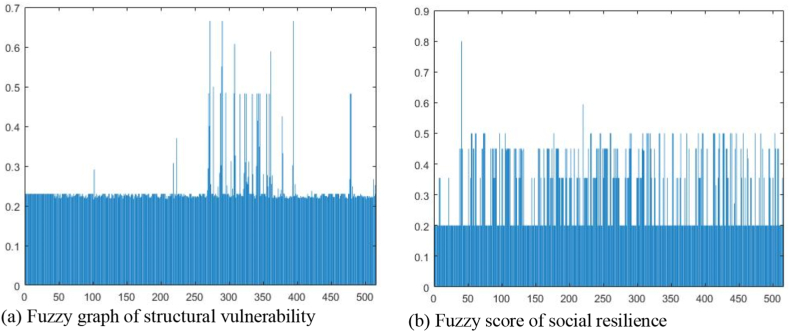
Fuzzy graph by lot number.

A high fuzzy score indicates high vulnerability and resilience, whereas a low fuzzy score indicates low vulnerability and resilience. As shown in Table 6, Table 7, Fig. 4, fuzzy values in the space unit of 100 m × 100 m grid were deduced using the standardized indicator values of vulnerability (Fig. 4a) and resilience (Fig. 4b).

**Table 6:** Standardiaztion and fuzzy score of structural vulnerability.

	Underground space	Building deterioration	Building material	Floor area ratio	Fuzzy Score
1	0.10616	0.03586	0.05556	0.02568	0.22426
2	0.15924	0.08311	0.16667	0.03859	0.21949
3	0.00010	0.05864	0.16667	0.00014	0.23000
4	0.01758	0.11020	0.06349	0.00498	0.22379
5	0.00010	0.00010	0.00010	0.00010	0.23000
6	0.11059	0.12150	0.13333	0.00010	0.23000
7	0.01980	0.21138	0.18391	0.15528	0.21968
:					
:					
512	0.00200	0.02870	0.34538	0.41107	0.26682
513	0.00210	0.03217	0.32864	0.32725	0.22435
514	0.00099	0.03329	0.45833	0.38967	0.25237
515	0.00053	0.00010	0.40000	0.36971	0.23000

**Table 7:** Standardiaztion and fuzzy score of social resilience.

	Land value	Working age population	Distance to medical and rescue facilities	Permeable area	Fuzzy Score
1	0.00010	0.00010	0.30319	0.00010	0.20000
2	0.00010	0.00010	0.47661	0.00008	0.20000
3	0.00010	0.34381	0.47661	0.00005	0.20000
4	0.00010	0.24778	0.47661	0.00008	0.20000
5	0.00010	0.27386	0.63258	0.00010	0.20000
6	0.00010	0.06995	0.63258	0.00008	0.20000
7	0.00010	0.12211	0.66354	0.00005	0.20000
:					
:					
512	0.21530	0.01838	0.72085	0.00005	0.20000
513	0.17912	0.02075	0.72085	0.00006	0.20000
514	0.10137	0.03260	0.72085	0.00005	0.35500
515	0.16061	0.01482	0.72085	0.00005	0.20000

### 3.4 Analysis of regional vulnerability and resilience

**Fig. 5:**
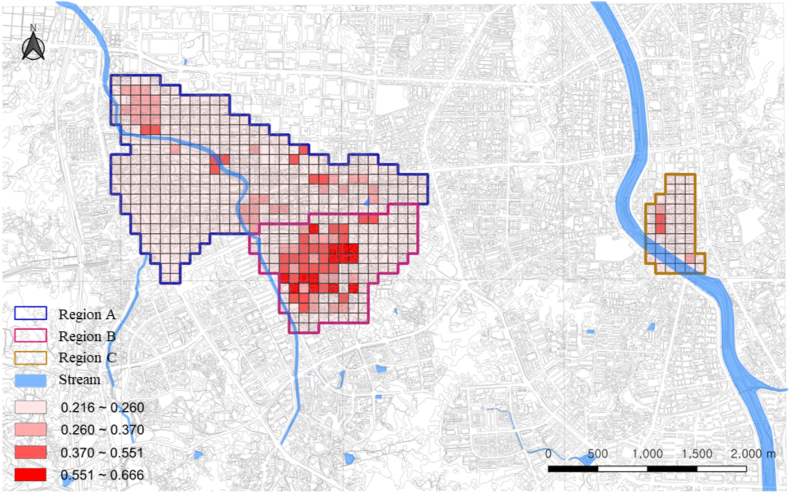
Regional analysis of structural vulnerability.

**Fig. 6:**
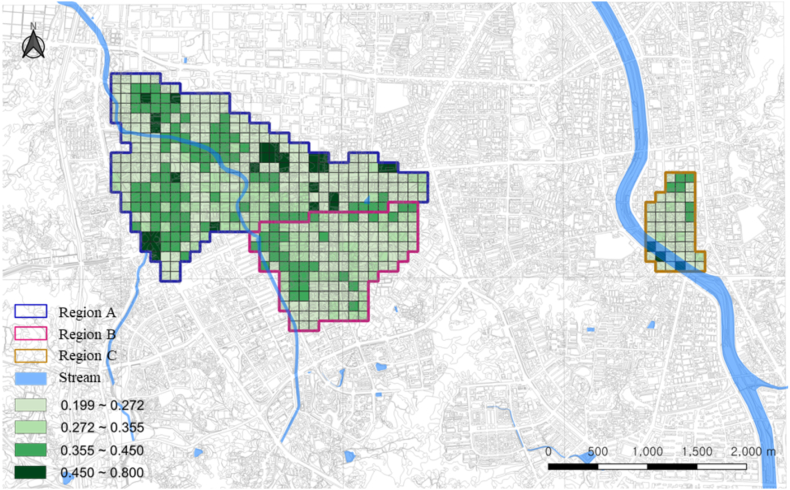
Regional analysis of social resilience.

Table 8, Fig. 5, Fig. 6 presents the regional vulnerability and resilience. The analysis results showed that “very high” and “high” vulnerability levels were the most prevalent in region B (26.95 %), followed by regions C (4.35 %) and A (2.74 %). Moreover, “very high” and “high” resilience levels were the most prevalent in region A (34.76 %), followed by regions B (21.28 %) and C (19.56 %).

**Table 8:** Structural vulnerability and social resilience rates by region.

		Fuzzy score	Highest		High		Moderate		Lowest	
			Cells of Grid Units	Ratio (%)	Cells of Grid Units	Ratio (%)	Cells of Grid Units	Ratio (%)	Cells of Grid Units	Ratio (%)
Structural vulnerability	Region A	0.2378	0	0.00	9	2.74	31	9.45	288	87.80
	Region B	0.3154	14	9.93	24	17.02	16	11.35	87	61.70
	Region C	0.2383	0	0.00	2	4.35	2	4.35	42	91.30
Social resilience	Region A	0.2973	28	8.54	86	26.22	49	14.94	165	50.30
	Region B	0.2268	0	0.00	30	21.28	37	26.24	74	52.48
	Region C	0.2690	3	6.52	6	13.04	5	10.87	32	69.57

Specifically, region A has low structural vulnerability and high social resilience, thus being relatively safer than other regions. Structural vulnerability focuses on geographically specific place or region [99,100]. Living in environments or spaces with low structural vulnerability and proper response capabilities improves stability [[101], [102], [103]]. According to Li et al. (2020), regions with proper response capabilities tend to have low possibility of experiencing disasters or accidents. For instance, regions with an earthquake response system can minimize human casualties and property damages caused by earthquakes [104,105]. In addition, evacuation and rescue efforts are smoothly carried out if regions have adequate response capabilities, thus enabling prompt and active response to accidents [106,107]. High social resilience also improves stability [108]. Social resilience refers to the ability to quickly recover from disasters or accidents combining various psychological and social elements [109]. High social resilience causes quick recovery from accidents, which ultimately minimizes casualties and property damages [110]. High economic level also allows safety measures to be established effectively [111,112]. Even when buildings are damaged, regular inspections and repairs can be performed or alternative residence can be found [113,114]. Furthermore, high social resilience minimizes the destruction of communities by disasters or accidents [115]. Thus, living in a place with low structural vulnerability and adequate response capability, in addition to high social resilience, can ensure improved stability [116,117].

Region B is deemed the most dangerous region since it demonstrates high structural vulnerability and low social resilience. People living in a region with extremely high structural vulnerability and extremely low social resilience belong to the most dangerous group in case of disasters. These individuals are often financially vulnerable, and have limited accessibility to resources and knowledge for sustaining a safe and healthy life [118,119]. During disasters, they face difficulties with being under the protection of structural stability, securing basic living necessities such as food and water, receiving medical services, establishing response strategies, or obtaining relevant information in real time. They required time and effort to recover from damages after a disaster [120,121]. Such circumstances develop the possibility of conflicts among neighbors or social exclusion from a community, and the aftermath of a disaster may last longer [122]. Therefore, people living in a structurally vulnerable region and having extremely low social resilience will face a high disaster risk.

Region C has both low structural vulnerability and social resilience, thus having somewhat adequate response capabilities. People who live in a city with structurally sound response capabilities but have low social resilience are highly likely to experience difficulties in overcoming or recovering from a disaster. This is because these people generally lack the ability to collect and handle necessary information, personal connections and resources to receive social support, and strong mentality to overcome difficulties when a disaster occurs [[123], [124], [125]]. For example, the reconstruction period of the socially vulnerable class after Hurricane Katrina hit New Orleans was significantly slower than that of other classes [126,127]. The socially vulnerable class had low accessibility to resources and information needed for reconstruction, in addition to insufficient networks for helping each other within local communities [128]. Therefore, even in regions with low structural vulnerability and adequate response capabilities, the socially disadvantaged group requires effective policy and support guaranteeing their safety and quality of life, as well as recognition of their hardships [129].

People with high social resilience and stable economic power can maintain safety and quickly recover from a disaster owing to adequate coping skills. However, those with low social resilience and inadequate response capabilities are more likely to receive serious damages from a disaster or an accident, thus requiring social protection. More efforts must be put forth to resolve structural vulnerability and improve social resilience.

### 3.5 Analysis of flood safety awareness

**Fig. 7:**
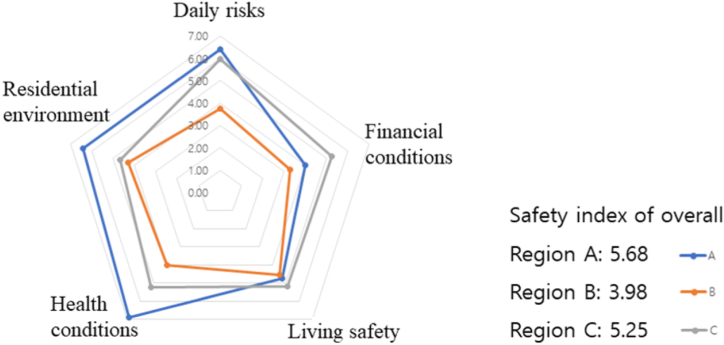
Safety awareness of flood damages.

The safety awareness concerning flood damages are shown in Table 9, Fig. 7. Region A recorded the highest overall safety awareness index (5.68), followed by regions C (5.25) and B (3.98). A high safety awareness index implies that individuals believe that the plans for preparation, prevention, and response to flood damages are well-established, while a low safety awareness index implies that the individuals perceive the risk of flood damage to a high extent. All three regions have experienced flood damages in the past, but their safety index are different due to their different social, economic, and infrastructure compositions.

**Table 9:** Safety awareness of flood damages.

	Daily risks	Financial conditions	Living safety	Health conditions	Residential environment	Safety index of overall
Region A	6.38	4.00	4.72	6.88	6.43	5.68
Region B	3.74	3.30	4.54	4.00	4.30	3.98
Region C	5.96	5.26	5.17	5.22	4.67	5.25

Specifically, the residents of Region A are relatively wealthier than those of Regions B and C, as it encompasses residential and commercial areas as well as a large shopping mall. The residents of Region A exhibited a comparatively higher level of flood safety awareness because a safe environment is emphasized in this main residential area. This result implies that flood response measures will be effective after exposure to a flood risk in Region A due to their heightened awareness and preparedness.

Region B demonstrated extremely low safety awareness, primarily because it includes mostly deteriorated residential areas. This signifies that structural defects and degraded durability in these areas heighten the flood risk. Residents in Region B are mostly socially vulnerable and perceive a high risk of floods; however, there is a lack of preparation and proper countermeasures. These regional circumstances and the low safety awareness of residents imply that more efforts and caution are needed to respond effectively to flood risks in Region B.

Region C, the old downtown with outdated facilities, is considered an economically affluent region. Since this region is the hub of economic activity, the residents are motivated to minimize economic damages from floods, and the high abundance of assets facilitates appropriate response and recovery measures. Despite the outdated infrastructure, the economic capacity of the residents enables them to implement effective flood responses.

In summary, the analysis results for the three regions demonstrate that flood safety awareness varies significantly by region. Establishing and managing proper flood responses, considering such regional characteristics are essential factors for strengthening the safety and resilience of a region. This study highlights the importance of tailored flood response strategies that account for the unique social and structural attributes of each region, thereby enhancing overall disaster preparedness and resilience.

Moreover, the concept of the 'vulnerability paradox' discussed by Verbong & Van der Vleuten (2004) is relevant here, as it highlights how regions with high economic activity and where infrastructure are taken for granted, might still exhibit vulnerabilities due to outdated infrastructure. Understanding and addressing these paradoxes is crucial for developing comprehensive flood management strategies [137].

## 4 Conclusion

This study proposed a new approach based on regional characteristics to address the issue of flood damages, which have recently become repetitive and massive because of the climate crisis. In particular, this study analyzed structural vulnerability, social resilience, and flood safety index to establish effective countermeasures based on regional characteristics. It is expected that diagnosing the current vulnerability and resilience of the study region regarding flood damages and enhancing the safety awareness of residents will help establish practical strategies needed for the region.

The findings showed that actual vulnerability and resilience exhibited different trends depending on regional characteristics. Region A had low structural vulnerability and high social resilience, region B had high structural vulnerability and low social resilience and region C had low structural vulnerability and high social resilience. These results provide critical information for identifying the unique vulnerability and resilience characteristics of each region. Region A has low structural vulnerability, which causes relatively less disaster damages, and high social resilience, which allows fast recovery. The flood safety awareness index of residents was also the highest. Region B has high structural vulnerability and low social resilience in addition to the lowest flood safety awareness index, thus urgently requiring comprehensive response measures. High structural vulnerability in Region C may cause certain damages, but its high social resilience allows for proper recovery and response measures.

In contrast to previous studies that primarily focused on financial support as a variable, our study provides a more comprehensive analysis of the correlation between social resilience and structural vulnerability. Emrich et al. (2020) highlighted that socially disadvantaged groups and minority households received less financial and recovery support despite severe damages, and other studies indicated that financial support was often inaccessible to minority and low-income groups. However, these studies did not sufficiently explore the broader correlation between social resilience and structural vulnerability.

Our study fills this gap by not only examining financial support but also considering a range of structural and social indicators. The results demonstrate that regions with different characteristics exhibit distinct patterns of vulnerability and resilience, which are closely linked to the flood safety awareness of residents. This comprehensive approach provides new insights into how these factors interact and affect regional preparedness and response capabilities.

Thus, the study results can be utilized for establishing individual response strategies by identifying the relationship between vulnerability and resilience in each region. This provides a guideline for effective actions during a disaster by deducing response measures considering regional characteristics. Furthermore, the results of the analysis of vulnerability and resilience concerning flood damages were similar to those of the flood safety awareness of residents, thus displaying a close interaction between the two aspects. This study is significant as it provides important information that can contribute to decision-making and regional policy proposals by validating the research purpose and expanding the understanding of the correlation between social resilience and structural vulnerability.

## CRediT authorship contribution statement

**Kiyong Park:** Data curation. **Seol A. Kwon:** Conceptualization.

## Data availability statement

Data will be made available on request.

## Funding statement

This research did not receive any specific grant from funding agencies in the public, commercial, or not-for-profit sectors.

## Declaration of competing interest

The authors declare the following financial interests/personal relationships which may be considered as potential competing interests:Seol A Kwon reports financial support was provided by 10.13039/501100002701Ministry of Education of the Republic of Korea. If there are other authors, they declare that they have no known competing financial interests or personal relationships that could have appeared to influence the work reported in this paper.
